# Time and Circumstances: Cancer Cell Metabolism at Various Stages of Disease Progression

**DOI:** 10.3389/fonc.2016.00257

**Published:** 2016-12-12

**Authors:** Georg F. Weber

**Affiliations:** ^1^James L. Winkle College of Pharmacy, University of Cincinnati, Cincinnati, OH, USA

**Keywords:** metabolism, therapeutics, metastasis, hypoxia, glycolysis

## Abstract

Over the past decade, research into the unique ways, in which cancer cells skew their metabolism, has had a renaissance—for the repeated time over more than 80 years since the discovery of an inherent preference for glycolysis. Importantly, the Warburg effect that arises in primary neoplasms is not the sole prominent metabolic phenomenon. Once the transformed cells are shed from their initial growth and begin the process of metastasis, their energy requirements change and they adapt to the increased demand for adenosine triphosphate, which if not satisfied would lead to anoikis. At that stage, oxidoreductases and the respiratory chain are activated. Furthermore, the intrinsic metabolic characteristics of tumor cells may be influenced by extrinsic factors, comprising metabolite secretions from stromal cells or acidification and nutrient deprivation in the late-stage hypoxic environment. While there is metabolic adjustment in cancer cells throughout the disease history, its phenotypic manifestation changes at various times. This stage selectivity has implications for pharmacotherapy ambitions.

## A Wealth of Information

Recent research has rediscovered the metabolic changes that occur in tumor cells during transformation. Numerous publications have studied the glycolytic preference of cellular energy pathways that is prevalent even under normoxic conditions. Yet, this so-called Warburg effect (the observation that cancer cells can grow with limited oxygen consumption by engaging glycolysis over mitochondrial respiration—even under conditions of sufficient oxygen supply) is not the only metabolic alteration that can arise in cancer cells. While some skewing of the metabolism seems to be inevitable in transformation, its presentation changes over the course of the disease. It is important to differentiate the various manifestations of cancer-associated metabolic adjustments (1).

The metabolic pathways within tumor cells can be altered endogenously, which is the case in the Warburg effect (the cells switch to glycolytic metabolism despite sufficient oxygen supply; if the switch occurs in a hypoxic environment, such as in advanced disease, it is not a Warburg effect, but an adaptation used by healthy and transformed cells alike). During tumor progression, when deadhesion occurs it poses a dramatically increased adenosine triphosphate (ATP) requirement that forces the upregulation of energy production. Alternatively, the microenvironment can affect the tumor cell metabolism when stromal cells secrete abundant lactate that is then taken up by the transformed cells. Obviously, the hypoxic, acidic, low-glucose environment of late-stage cancers also has a direct effect on the metabolic processes inside the cancer cells. The connotations of these four scenarios (intrinsic, deadherent, stromal-induced, and late-stage) differ among each other in underlying mechanisms and resulting phenotypes (Table 1). Consecutively, they require distinct strategies for ambitions to incorporate metabolic targeting into anticancer treatment regimens.

**Table 1:** **Stage-dependent alterations in cancer cell metabolism**.

	Cause	Effect	Potential treatment
**Intrinsic (Warburg)**	P53-deficient tumor cells do not have functional SCO2 or TIGAR and display a glycolytic metabolism phenotype	Protect from apoptosis by closing Kv channels and preventing the influx of calcium	Dichloroacetate, lonidamine
	Embryonic M2 isoform of pyruvate kinase shifts cellular metabolism to aerobic glycolysis	Satisfaction of anabolic requirements, biosynthetic activities by proliferating tumor cells entail the production of ribose-5-phosphate for nucleotide biosynthesis, and the production of fatty acids for lipid biosynthesis	

**Deadhesion**	Deadherent cells suffer deficit in glucose transport, resulting in adenosine triphosphate (ATP) deficiency and anoikis	Peroxide signaling, increased mitochondrial activity	Anti-oxidants
		Serine–glycine–creatine pathway regenerates ATP	

**Stromal interaction**	Lactate secretion from mesenchymal cells *via* the transporter MCT4	Tumor cells import this lactate *via* MCT1 expression, converting it to pyruvate and introducing it into the Krebs cycle, resulting increased in oxidative phosphorylation and ATP production	

**Hypoxia**	Hypoxia, low-glucose, lactate	Induction of HIF-1, carbonic anhydrase IX	Methazolamide

## Primary Growths: Warburg’s Glycolysis

The evolution of respiration has equipped us with a tremendous advantage. The oxidative breakdown of glucose, the fuel in bioenergetics, yields 36–38 molecules of ATP per molecule of glucose, as opposed to 4–6 molecules of ATP resulting from glycolysis. Initially, it seemed paradoxical that tumors, which need more chemical energy than healthy cells due to their rapid proliferation, would preferentially engage the much less efficient glycolytic energy production. Two fundamental questions need to be addressed to understand this Warburg effect (aerobic glycolysis). What causes it and what are its consequences?

Various explanations have been put forward for the molecular causes of glycolysis in cancer (2, 3). In healthy cells, P53 supports ATP generation by stimulating oxidative phosphorylation (through the activation of SCO2) and inhibiting glycolysis (through the inactivation of PFK-1). P53 directly regulates oxidative phosphorylation by stimulating the expression of the gene that encodes SCO2, which is essential for the assembly of the cytochrome *c* oxidase complex embedded in the inner mitochondrial membrane. The P53-inducible gene TIGAR codes for a protein that downregulates the cellular fructose-2,6-bisphosphate levels. As fructose-2,6-bisphosphate is an allosteric effector of 6-phosphofructose-1-kinase (PFK-1), which promotes glycolysis, TIGAR expression attenuates this pathway. Loss of P53 function is common in human cancers. TP53-deficient tumor cells do not have functional SCO2 or TIGAR and display a glycolytic metabolism phenotype (4, 5). In addition, functional gains may be exerted by TP53 mutations that commonly arise in cancer. Mutated P53 stimulates the Warburg effect through promoting the translocation of the glucose transporter GLUT1 to the plasma membrane. This is mediated by activated RHO-A and its downstream effector ROCK (6). It is conceivable that the abundance of intracellular glucose requires its processing *via* glycolysis rather than *via* the slower oxidative phosphorylation.

Tumor cells express exclusively the embryonic M2 isoform of pyruvate kinase, which is necessary for the shift in cellular metabolism to aerobic glycolysis. This switch in a splice variant of the glycolytic enzyme allows these cells to proliferate in low glucose and limiting oxygen conditions that are common in cancer. The division of cells expressing the M1 variant is significantly decreased compared to M2 expressing cells in low oxygen (of note, this characteristic reflects hypoxia resistance, which is not identical to the Warburg effect). Furthermore, pyruvate kinase M2 expression provides a selective growth advantage for tumor cells *in vivo* (7).

## Warburg’s Effects

The reliance by rapidly growing cancer cells on anaerobic glycolysis seems counterintuitive. Hence, teleological explanations for why and how the preferred glycolytic metabolism in transformation may advance tumor initiation or tumor growth have been scant. Two possible justifications associate cancer cell glycolysis either with mitochondrial hyperpolarization and anti-apoptosis or with the synthesis of essential biomolecules. In some cancers, the aerobic glycolysis may support oncogenic transcription.

The cell membrane contains a voltage-gated family of potassium channels (Kv), which due to its redox sensitivity can be regulated by the mitochondria. As a by-product of respiration, the mitochondria produce superoxide, which may be dismutated to hydrogen peroxide that activates these channels, thereby regulating the voltage-dependent influx of calcium and the activity of caspases. The connection of mitochondria *via* peroxide to Kv channels is involved in oxygen sensing as well as in the promotion of apoptosis. Cancer cells would be at risk of cell death if the rapid proliferation relied on energy production by the mitochondria, thus generating high levels of superoxide and—derived from it—hydrogen peroxide. However, mitochondrial hyperpolarization occurs in these transformed cells and leads to anti-apoptosis, because low hydrogen peroxide and high NFAT suppress the plasma membrane potassium channels (8).

Among the genes that can initiate tumorigenesis, many are closely linked to metabolic regulation. In this context, the biosynthetic processes required to divide and create daughter cells are equally important for tumor growth as is bioenergetics. Because of the preference for aerobic glycolysis in cancer cells, the glucose-derived metabolite feed into the tricarboxylic acid cycle is reduced. Thus, cancer cells typically have an increased reliance on alternative biomolecules to replenish Krebs cycle intermediates, and the amino acid glutamine is such a metabolite (9). The anabolic prerequisites for these pathways are met if the glutamine metabolism generates NADPH, which restores oxaloacetate. Two necessary biosynthetic activities by proliferating tumor cells entail the production of ribose-5-phosphate for nucleotide biosynthesis and the production of fatty acids for lipid biosynthesis. The prevalent glutaminolysis in transformed cells enables the use of glucose carbon for lipid, protein, and nucleotide synthesis (10, 11). This model is supported by observations that several signaling pathways implicated in cell proliferation also regulate metabolic pathways that incorporate nutrients into biomass and that certain cancer-associated mutations enable the cells to acquire and metabolize nutrients in a manner conducive to anabolism rather than to efficient ATP production (12).

The transcription factors YAP and TAZ, mediators of the Hippo pathway, promote organ growth, tumor cell proliferation, and cancer aggressiveness. When cells actively incorporate glucose and route it through glycolysis, YAP/TAZ transcription is fully active. When glucose metabolism is blocked or glycolysis is reduced, YAP/TAZ activity is decreased. Accordingly, glycolysis is required to sustain YAP/TAZ pro-tumorigenic functions, and YAP/TAZ is required for the full deployment of glucose growth-promoting activity (13).

## Travelers: The Metabolism of Deadherent Cells

The dissemination of transformed cells is an integral characteristic of cancers, but it is absent from benign growths. Metastases manifest clinically at advanced disease stages. While the major limiting factor in cancer spread is the death of the tumor cells before their implantation into target organs (14–16), a fraction of the released malignant cells can survive in the circulation for extended periods of time. The molecular programs of metastasis act to promote tumor progression, not growth or extension of life span (17, 18). Besides inducing directed migration and invasion, they support adhesion-independent survival, which is more critical to the process of cancer metastasis than organ-specific homing (19). An excess of anchorage independence is pathogenic in cancer spread.

To accomplish survival after deadhesion, metastasis genes affect metabolic adjustments that are distinct from the Warburg effect. Untransformed non-hematopoietic cells (with the exception of tissue-resident stem cells) undergo anoikis consecutive to losing contact with their substratum. In these healthy cells, anchorage deprivation causes an impairment in glucose transport, a deficit in ATP (i.e., in chemical energy) and consecutive programed cell death (anoikis) (20). Cancer cells that have been released from a primary tumor need to overcome the energy shortfall to survive and form metastases. Consistent with these requirements, increased cancer invasiveness under anchorage-deprived conditions is associated with higher mitochondrial activity, elevated ATP production, pyruvate uptake, and oxygen consumption (21).

In cancer cells that have been shed from the initiating neoplasm, the gene products of metastasis support enhanced energy generation, manifested in elevated ATP synthesis. Biochemical processes associated with the mitochondria may satisfy an increased energy requirement once these cells lose contact with the substratum. Variant forms of the cytokine osteopontin act as autocrine inducers. Osteopontin is a metastasis gene product that supports the progression of over 30 malignancies (22, 23). The protein exists in three splice variants, dubbed osteopontin-a, -b (lacking exon 5), and -c (lacking exon 4) (19). The variants osteopontin-b and -c have only been observed in transformed cells, and they are never expressed without the full-length gene product osteopontin-a. The distinct splice forms may synergize in support of anchorage-independent survival. Osteopontin-a increases the levels of glucose in deadherent cells. Signaling *via* osteopontin-c upregulates peroxides as well as intermediates of the hexose monophosphate shunt and glycolysis, which utilize the available glucose and can feed into the tricarboxylic acid cycle. Consecutively, the cellular ATP levels are elevated (24, 25). The role of the cancer-specific splice variants may account for the inability of non-transformed cells (which do not splice the osteopontin RNA) to overcome anoikis.

Elevated levels of hydrogen peroxide mediate metabolic changes that allow increased energy production, deadherent survival, and consecutive metastatic spread. Whereas research has widely focused on the potential pro-apoptotic functions of hydrogen peroxide (also note one of the teleological models for the Warburg effect), a growing literature that describes peroxide as essential for cancer metastasis (1) has received less attention. Anchorage-independent expansion is supported by peroxide signaling (26–28), which is tied to ATP generation, albeit through incompletely elucidated mechanisms. A hypothesis suggests that hydrogen peroxide inhibits anoikis through the suppression of caveolin-1 ubiquitination and degradation. Caveolae–mitochondria interaction regulates the adaptation to cellular stress by modulating the structure and function of the mitochondria. Through this mechanism, caveolin-1 is a key protein involved in tumor metastasis. An alternative hypothesis implies that ATP-sensitive potassium channels (K_ATP_ channels) can be activated by hydrogen peroxide in the mitochondrial membrane and exert anti-apoptotic effects, thus linking bioenergetics and survival. Either one of these interactions (potassium channels or caveolin) could account for the oxidative effects of osteopontin-c.

Anti-anoikis signals may be transduced *via* peroxides. The mitochondria generate reactive oxygen species, predominantly through their complex III. These are required for K-RAS-mediated anchorage-independent growth, which is accomplished *via* regulation of the ERK/MAPK signaling pathway (29). Escape from anoikis through the production of reactive oxygen species can also be mediated by oxidation and activation of the tyrosine kinase SRC, which results in the transduction of a survival signal (30). While the upregulated oxidoreductases are confirmed important mediators of deadherent survival, the modalities by which the K-RAS and SRC pathways may affect metabolism in cancer cells are only now being elucidated. K-RAS may induce a non-canonical, but essential pathway of glutamine use in pancreatic cancers. Whereas most healthy cells use glutamate dehydrogenase to convert glutamine-derived glutamate into α-ketoglutarate in the mitochondria, pancreatic cancer cells transport glutamine-derived aspartate into the cytoplasm, where it is converted *via* oxaloacetate and malate, the further metabolism of which ostensibly increases the NADPH/NADP+ ratio required to maintain the cellular redox state (31).

## ATP Regeneration: The Serine–Glycine–Creatine Pathway

Some of the metabolites that are upregulated by osteopontin-c, such as serine, glycine, and glycerol, are typically elevated in aggressive cancers. Glycine pathways may contribute to both, tumor growth and cancer dissemination. These molecules have emerged as potential targets for anticancer treatment strategies.

**Figure 1:**
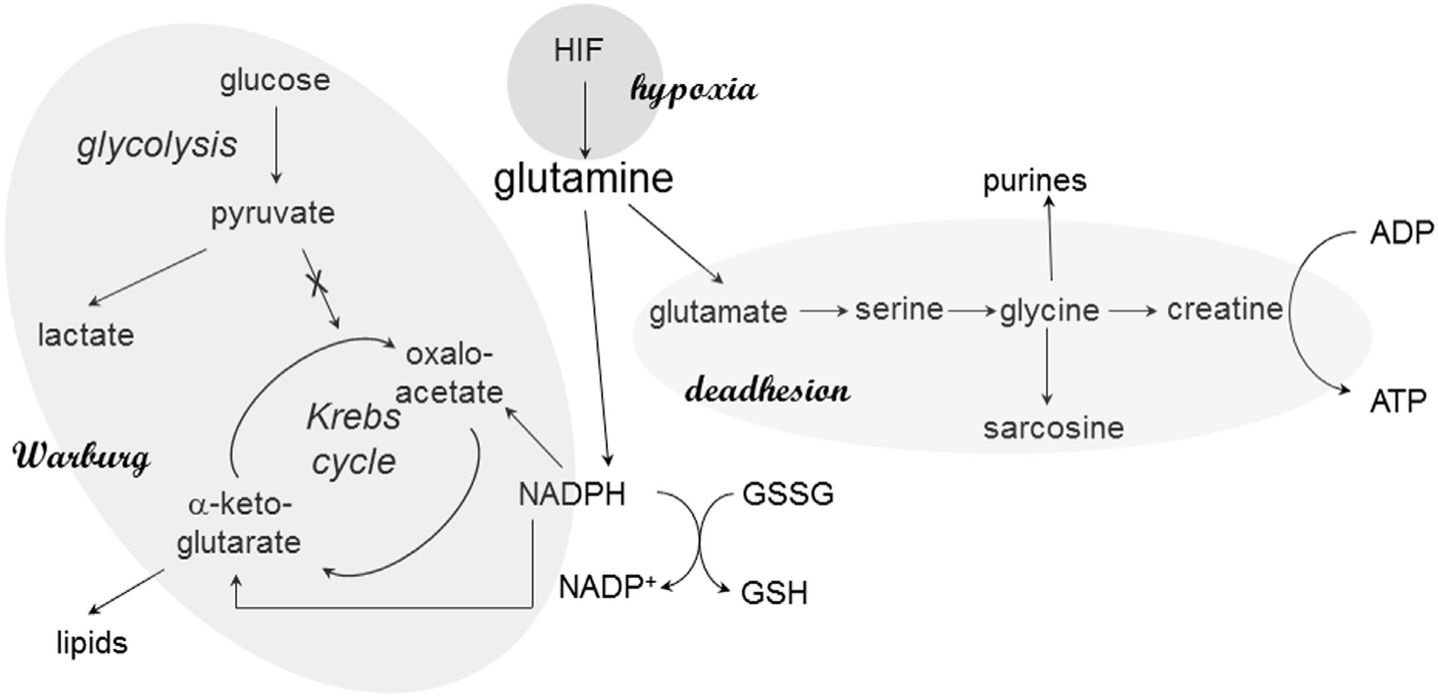
**Glutamine in cancer cell metabolism**. At various stages, glutamine plays critical roles in the metabolic skewing within cancer cells. The reason may be its central place in pathways associated with Warburg’s anabolism (*via* NADPH), energy generation during deadhesion, and hypoxic responses. Through its connection to NADPH production, glutamine also maintains the cellular redox balance *via* glutathione regeneration.

Adenosine triphosphate can be generated *via* the respiratory chain or *via* glycine and creatine in deadherent cells. The glycine pathway is frequently upregulated in cancer progression; in breast cancer, the splice variant osteopontin-c is the inducer (25). Osteopontin-c signaling increases the levels of glutamate and glycine as well as its metabolic product creatine, which may regenerate cellular ATP independently of the mitochondria. The elevated production of glycine and ensuing ATP regeneration *via* creatine supports anchorage-independent cell survival, in part through an increase in glutaminolysis (Figure 1). Furthermore, glycine may form sarcosine *via N*-methylation. Sarcosine is a metabolite that is highly elevated during prostate cancer progression to metastasis (32).

Glycine consumption and expression of the associated mitochondrial biosynthetic pathway are strongly correlated with the rates of proliferation in diverse cancer cells (33, 34). Even though glycine is a non-essential amino acid, which can be endogenously synthesized, the demand for it may exceed the endogenous synthesis capacity in rapidly proliferating cancer cells. By contrast, in slowly proliferating cells, glycine synthesis may exceed the demand. The purpose for the amino acid is twofold. Glycine is utilized for *de novo* purine nucleotide biosynthesis in some rapidly proliferating cells. Utilization of one-carbon groups derived from glycine for cellular methylation reactions may be important in other cancer cell types.

## Group Dynamic: Metabolic Cell–Cell Interactions

Stromal cells that surround a tumorous growth may enable the acquisition of a pro-invasive metabolic profile in the cancer cells. Despite aerobic conditions, glycolysis may be induced in mesenchymal stem cells that are in proximity to osteosarcoma cells. These untransformed cells then secrete lactate *via* the transporter MCT4. The tumor cells import this lactate because of their MCT1 expression, converting it to pyruvate and introducing it into the Krebs cycle. Thus, oxidative phosphorylation and ATP production are increased in osteosarcoma cells by surrounding mesenchymal stem cells *via* oxidative stress. This mechanism enhances their aggressive behavior (35).

The levels of P62 are reduced in the stroma of various tumors. This loss in the stromal fibroblasts results in increased tumorigenesis of epithelial prostate cancer cells through the regulation of the cellular redox balance. Underlying is an mTORC1/c-Myc pathway of stromal glucose and amino acid metabolism, which causes increased stromal IL-6 production that is required for tumor promotion. Thus, P62 is an anti-inflammatory tumor suppressor that acts through the modulation of metabolism in the tumor stroma (36).

## The Late Stage

During early stages of transformation, tumor cells acquire gain-of-function mutations in oncogenes or loss-of-function mutations in tumor suppressor genes that cause excessive proliferation and anti-apoptosis. As the transformed cells multiply, they outgrow the diffusion limits of oxygen, thus, becoming hypoxic. Because of increased glycolysis, more lactic acid is generated, which makes the lesions acidic. Even though new blood vessels are formed in cancer angiogenesis, they are disorganized and cannot effectively alleviate this state. One of the important pathophysiological properties of advanced stage tumors is the prevalence of lactacidosis, hypoxia, and low glucose.

Human cells, non-transformed and transformed, are specifically equipped to sense the available oxygen in their microenvironment and respond to changes. The hypoxia-inducible transcription factors HIF-1 and HIF-2 coordinate the adaptive cellular response to low-oxygen tension. Under normoxic conditions, prolyl hydroxylases use oxygen as a substrate to hydroxylate key proline residues within the α subunits of HIF-1 and HIF-2. This mechanism allows the tumor suppressor VHL (an E3 ubiquitin ligase) to target these α subunits for proteasomal degradation, thus preempting a low-oxygen response. Under hypoxic conditions, prolyl hydroxylase activity is suppressed, resulting in HIF-α stabilization and translocation to the nucleus. There, HIF-α subunits dimerize with aryl hydrocarbon receptor nuclear translocator, recruit transcriptional coactivators such as p300/CBP, and bind to hypoxia-responsive elements in target genes to activate transcription. They induce gene expression programs, which regulate glucose uptake and metabolism to affect proliferation and survival.

Once a cancer mass has grown to a certain size, the genetic programs of transformation act in a non-conducive environment. The characteristic metabolic conditions of advanced tumors (lactacidosis, hypoxia, and low glucose) activate unique intracellular signals (37). Because glycolysis constitutes a common metabolic pathway in cancer cells that leads to the generation and accumulation of high levels of lactic acid, their intracellular pH drops substantially. In this setting, the cancer cells maintain their homeostasis in part through the actions of carbonic anhydrases. These enzymes catalyze the reversible hydration of carbon dioxide and thus contribute to pH maintenance. Carbonic anhydrase IX (CAIX) is a membrane-bound enzyme that catalyzes the conversion of water and carbon dioxide to bicarbonate ions and protons, extracellularly. These bicarbonate ions are then transported inside the cells, elevating the intracellular pH toward physiologic levels, so that cell survival is assured. Through the same process, CAIX leads to an accumulation of protons extracellularly, which makes the microenvironment more acidic. Acidification of the extracellular space could support cancer cell motility and increase invasion with resulting metastasis formation. CAIX may be over-expressed in hypoxic cancer cells as a result of increased glycolysis and acidic pH. The enzyme is a downstream mediator of HIF-1α, which is activated by hypoxia.

HIF activity in hypoxic cells promotes the conversion of glucose to lactate, thus preventing its utilization in the Krebs cycle. A loss of HIF regulation by VHL, and resultant HIF hyper-activation, is sufficient to switch the input onto the Krebs cycle from mostly glucose derived to glutamine derived. Intracellular citrate deficiency may promote this switch. Cancer cells in this state use glutamine to generate citrate and lipids through the reductive carboxylation of glutamine-derived α-ketoglutarate. Consistently, VHL deficiency sensitizes cancer cells to the inhibition of glutaminase, the enzyme that catalyzes the first step of glutamine metabolism (9).

## The Eyes of the Researcher

**Figure 2:**
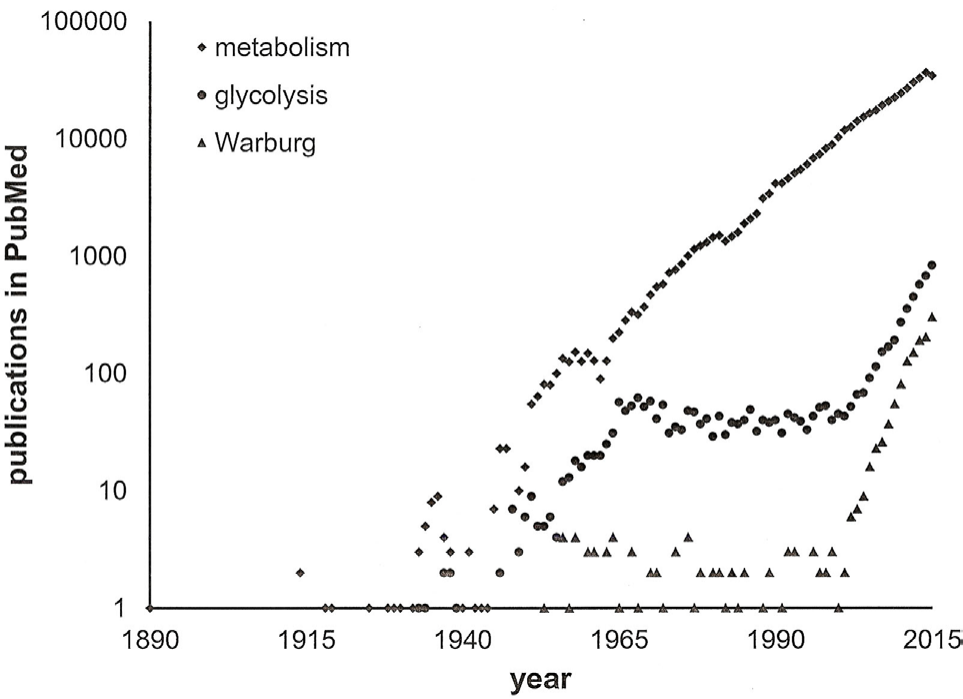
**Publications on cancer metabolism**. A search in PubMed with the keywords “cancer” and either “metabolism” or “glycolysis” or “Warburg” through 2015 indicates the recent surge in activity within the field. Note the logarithmic scale of the *y*-axis.

We have learnt that the intermediary pathways for energy generation are skewed in cancer cells, such that they favor aerobic glycolysis in primary tumors and stimulation of ATP generation in disseminating cancer cells. High-profile publications on glycolysis versus oxidative respiration have attracted scientists in the field and have stimulated additional research into energy pathways. Because the stage was set, the same core pathways that had been pinpointed early on have been investigated over and over (Figure 2). The self-amplifying way, in which the research has been conducted, may have attributed disproportionate importance to glycolysis and its immediately connected metabolic processes. While more recent studies have ventured toward the analysis of branching pathways (such as the serine–glycine–creatine connection), energy generation has been the broad area of interest. Newer theories have raised the possibility that anabolism may equally importantly be adjusted in cancer cells (11). It is conceivable that pathophysiology—specifically in cancer—alters the metabolism more extensively than is thus far known. Research may need to expand its focus from glucose utilization and energy generation to the processing of other sugars and lipids, to the homeostasis of NADH and NADPH, and to the biosynthesis of nucleic acids, proteins, and other cellular components.

Besides the major energy source glucose, transformed cells can use alternative energy supplies. Non-glycolytic pathways are operational in cancers even under hypoxic conditions. Among them, fatty acid oxidation may be a dominant bioenergetic pathway in prostate cancer cells (38). Fatty acid synthase, a key enzyme of the lipid metabolism, is upregulated in many cancers. Drug- and radiation-resistant tumor cells use fatty acid to support mitochondrial oxygen consumption when glucose becomes limited. Specific fatty acid synthase inhibitors may possess anti-tumor activity.

**Figure 3:**
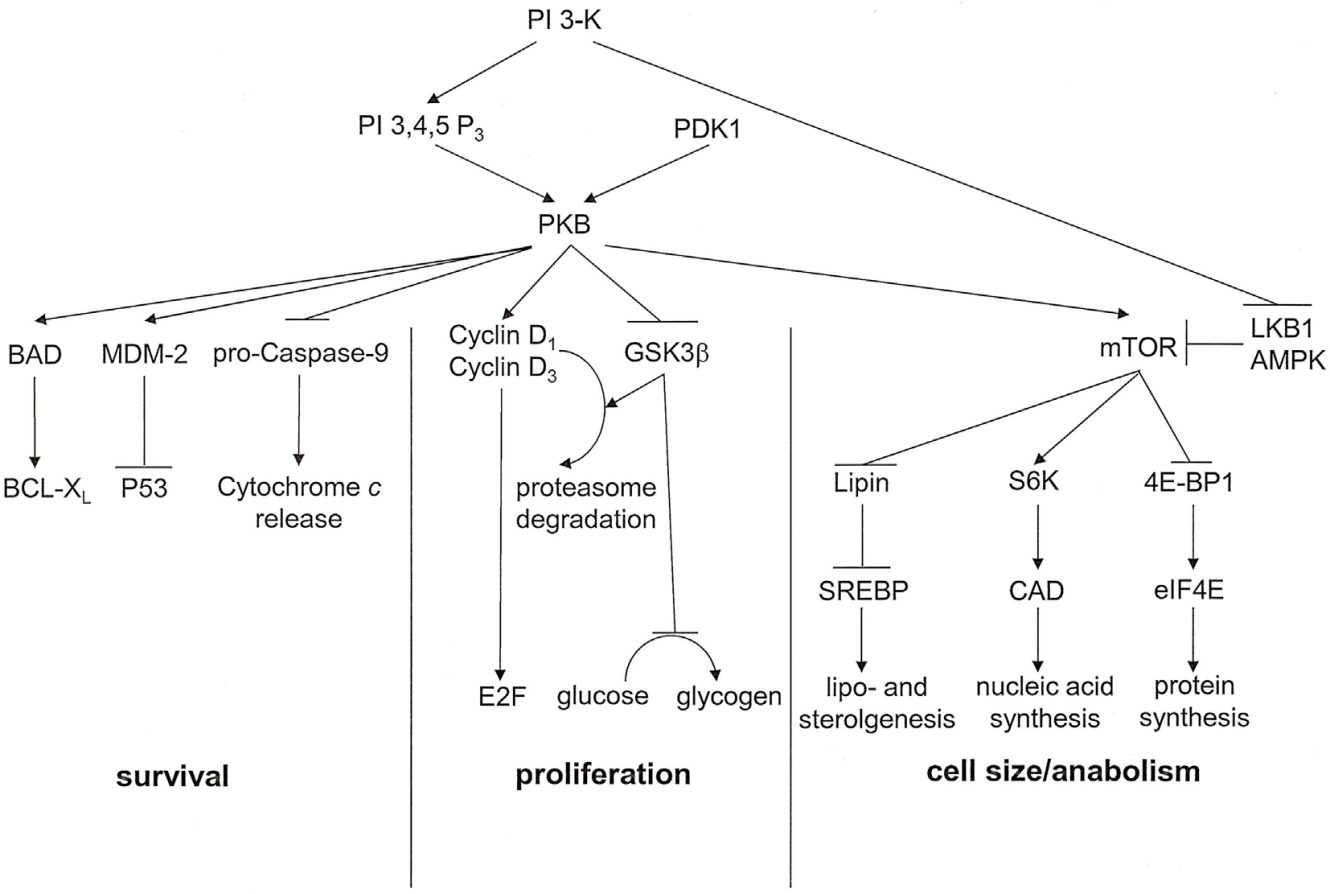
**PKB associated signal transduction pathways**. PKB is an essential mediator of the lipid kinase signal transduction pathway. It exerts effects that lead to cell cycle progression, cell survival, and regulation of cell size. For each of these outcomes, multiple signaling intermediates synergize to induce the resultant biological effect. PI 3,4,5 P_3_, phosphatidylinositol 3,4,5-trisphosphate [adapted from Ref. (17)].

Activated *via* receptors for PDGF, insulin, MET, and CSF-1, the proto-oncogene product phosphatidylinositol 3′-kinase (PI 3-K) has important metabolic functions (Figure 3). It catalyzes the synthesis of phosphatidylinositol 3,4,5-trisphosphate or phosphatidylinositol 3,4-bisphosphate, which are ligands for Plekstrin homology domains in various proteins. It also associates with and activates the proto-oncogene product PKB. Ensuingly, PKB signaling plays key roles in cell cycle progression, cellular survival, and managing increased cell mass. The downstream target of this cascade, mammalian Target of Rapamycin (mTOR), is a serine/threonine kinase that oversees cell growth and metabolism in response to growth factors and nutrients. mTOR also senses oxygen and energy levels. It acts as a central regulator of cell size. mTOR controls biogenesis based on the availability of nutrients by activating RSK (P70 ribosomal S6 kinase), which enhances the translation of mRNAs that have 5′ poly-pyrimidine tracts. PI 3-K inhibits the tumor suppressing serine–threonine kinase LKB1, which is frequently lost in cancers, especially in lung cancers. LKB1 links cell metabolism to growth control and cell polarity. It phosphorylates and activates the central metabolic sensor AMP-activated protein kinase (AMPK). AMPK is a metabolic switch that governs glucose and lipid metabolism in response to alterations in nutrients and intracellular energy levels. The kinase controls anabolic pathways related to cell growth (39). The PI 3-K pathway has gained attention as a potential target for metabolic and anti-proliferative treatment in patients with cancer.

The mevalonate pathway may control YAP/TAZ transcriptional activity *via* its rate-limiting enzyme, HMG-CoA reductase. The geranylgeranyl pyrophosphate produced by the mevalonate cascade is required for the activation of RHO GTPases that activate YAP/TAZ. In tumor cells, the oncogenic cofactor mutant P53 induces SREBP transcriptional activity, which causes increased levels of mevalonate and consecutive activation of YAP/TAZ. The expression of receptor for hyaluronan-mediated motility (RHAMM) is regulated by the convergence of mevalonate and Hippo pathways onto YAP/TEAD, which controls RHAMM transcription and consequently supports breast cancer cell migration and invasion. Expression of the breast cancer susceptibility gene RHAMM is tightly controlled in healthy tissues but elevated in many tumors, contributing to tumorigenesis and metastases (40, 41).

## Implications for Cancer Therapy

The characteristics of transformed cells have generated an opportunity to target their metabolism with drug treatment, a concept referred to as metabolic intervention (42). Existing regimens have concentrated on the Warburg effect, hypoxia or the PI 3-K, and mevalonate pathways.

### Warburg Effect

Because of metabolic and mitochondrial defects, tumor cells often preferentially use glycolysis to generate ATP, even in the presence of oxygen, the phenomenon known as the Warburg effect. Dichloroacetate (DCA, Ceresine) is an inhibitor of mitochondrial pyruvate dehydrogenase kinase, which inhibits pyruvate dehydrogenase, a gatekeeping enzyme for the entry of pyruvate into the tricarboxylic acid cycle. DCA treatment may reactivate mitochondrial respiration in tumor cells, induce their selective killing, and suppress cancer growth (8). The preferential targeting of cancer cells by this agent occurs through two mechanisms. DCA shifts the metabolism toward aerobic respiration, such that depolarization of the mitochondrial membrane induces the release of pro-apoptotic factors. DCA promotes increased hydrogen peroxide generation, which activates potassium channels. Kv1.5 inhibits the calcium-dependent transcription factor NFAT, which impairs apoptosis. The activation of Kv1.5 may decrease cellular potassium, thus activating caspases and triggering apoptosis.

Dichloroacetate is used to treat hyperglycemia in diabetes mellitus, because it stimulates peripheral glucose utilization and inhibits gluconeogenesis through its effect on pathways of the intermediary metabolism. By decreasing circulating lipid and lipoprotein levels, it suppresses lipogenesis and cholesterolgenesis in patients with acquired or hereditary disorders of the lipoprotein metabolism. By stimulating the activity of pyruvate dehydrogenase, DCA facilitates the oxidation of lactate and decreases morbidity in acquired and congenital forms of lactic acidosis. The drug is dehalogenated to monochloroacetate and glyoxylate, from which it can be further catabolized to glycolate, glycine, oxalate, and carbon dioxide. At sustained, higher doses, there is an increased risk of neurotoxicity and gait disturbance. DCA can cause a reversible peripheral neuropathy that may be related to thiamine deficiency and may be ameliorated or prevented with thiamine supplementation. At high doses, the drug itself can be carcinogenic.

The indazole carboxylate lonamidamine suppresses aerobic glycolysis in cancer cells but enhances it in untransformed cells, likely through the inhibition of mitochondrial hexokinase. This causes a reduction in cellular ATP levels. The drug may also act as a putative ligand for adenine nucleotide translocator (exports ATP from the mitochondrial matrix and imports ADP into the matrix) that triggers apoptosis. Lonidamine is in clinical trials for the treatment of brain tumors.

### Hypoxia

Distinct from the Warburg effect, late-stage tumors often incur hypoxia because they have outgrown their blood and oxygen supply. As a hypoxia-inducible transmembrane glycoprotein, CAIX catalyzes the rapid interconversion of carbon dioxide and water into carbonic acid, protons, and bicarbonate ions, helping to maintain acidification of the tumor microenvironment and enhance resistance to cytotoxic therapy in some hypoxic tumors. Methazolamide (*N*-[5-(aminosulfonyl)-3-methyl-1,3,4-thiadiazol-2(3H)-ylidene]-acetamide) is a sulfonamide derivate that inhibits tumor-associated CAIX (43). Thus, it may cause increased cell death in hypoxic tumors. Common adverse reactions, occurring most often early in therapy, include paresthesias, tinnitus, fatigue, malaise, loss of appetite, taste alteration, gastrointestinal disturbances (nausea, vomiting, and diarrhea), polyuria, and occasional instances of drowsiness and confusion. The drug can cause metabolic acidosis and electrolyte imbalance. Transient myopia subsides upon diminution or discontinuance of the medication. Rare but dangerous adverse reactions to sulfonamides include toxic epidermal necrolysis (Stevens–Johnson syndrome), fulminant hepatic necrosis, agranulocytosis, or aplastic anemia. Methazolamide should be used with caution in patients on steroid therapy because of the potential for developing hypokalemia. Under concomitant use of high dose aspirin, carbonic anhydrase inhibitors can lead to anorexia, tachypnea, lethargy, coma, or death.

### PI-3-Kinase Pathway

In many cancers, the phosphatidylinositol 3′-kinase pathway is upregulated, either by elevated levels of insulin or IGF or by loss-of-function mutations of the tumor suppressor PTEN. Obesity and diabetes are accompanied by increased cancer risk, which may be due to high circulating levels of the growth factors insulin and IGF. Diabetics treated with metformin have 25–40% reduced incidence of cancer compared to those who receive insulin as therapy or take sulfonylurea drugs that increase insulin secretion from the pancreas. Metformin activates the enzyme AMPK in the liver, which then reduces the synthesis and secretion of glucose, thereby lowering the blood glucose levels. The drug also downregulates the blood insulin and IGF levels. Furthermore, metformin stimulates the tumor suppressor gene LKB1. Due to these properties, metformin is under investigation for cancer treatment or prevention.

### Mevalonate Pathway Inhibitors

Statins are pharmacologic inhibitors of HMG-CoA reductase. They are used as lipid-lowering medications. Statins reduce cardiovascular disease and mortality in high-risk patients. The class of drugs includes atorvastatin, fluvastatin, lovastatin, pitavastatin, pravastatin, rosuvastatin, and simvastatin. Adverse effects may comprise muscle pain, increased risk of diabetes mellitus, liver damage, and rare but severe muscle damage. As HMG-CoA reductase is the rate-limiting enzyme of the mevalonate pathway, statins have potential application in anticancer treatment to inhibit YAP/TAZ. While the literature is not conclusive on the subject, there are reports of reduced risk for certain cancers under statin therapy.

## Prospects

The observation that cancer cells alter their metabolism compared to healthy cells has been firmly established. More recent research has dissected various phenotypic manifestations of the metabolic changes according to intrinsic, deadherent, stromal-induced, or late-stage conditions. Despite this progress, it is remarkable how many gaps in knowledge still exist. The pathophysiological purpose of the Warburg effect is incompletely understood. Hypotheses to explain the mechanism, through which peroxide signaling increases the cellular ATP levels after deadhesion, are yet untested. Pathways outside hexose monophosphate shunt, glycolysis, and tricarboxylic acid cycle have not been subjected to extensive research. While every new insight into cancer generates a desire to derive therapeutic applications, the limited understanding of the multiple molecular connections compromises the development of efficacious strategies.

Historically, the challenge for anticancer therapy has been the lack of qualitative differences between tumor cells and host cells. In infectious diseases, antibiotic therapy has been so successful because it targets molecules of the pathogens that are highly distinct from humans, thus achieving very manageable adverse effects. As cancer cells are derived from self, this benefit was long not available to anticancer drug treatment. Therefore, conventional chemotherapy drugs, which rather non-specifically target cell division or DNA synthesis, have been fraught with high toxicity (predominantly to the rapidly proliferating cells in the immune system, the gastrointestinal tract, and the hair) and moderate efficacy due to inevitable dose limitations. One of the major accomplishments in translating molecular biology research into clinical applications has been the targeting of mutated molecules that are causative and specific for cancer cells, including BCR-ABL, mutated EGFR, and HER2. Similarly, anti-metastasis drugs neutralize molecules that are rarely expressed in the adult healthy organism, such as VEGF or integrin α_V_β_3_. The targeting of altered metabolic pathways has its focus on quantitative, not qualitative, differences between tumor and host cells. While it is unlikely that such a modality could stand on its own, it is conceivable that anti-metabolism treatments may be added to other cancer therapies and may synergize in combination regimens. It will be important to bear in mind the metabolic differences among primary tumors (Warburg effect), metastasizing cancer cells (peroxide-driven ATP production), tumor cells under stromal influence (lactate uptake, inflammation), and late-stage cancers (hypoxia) so as to direct anti-metabolism agents safely and efficaciously.

## Author Contributions

The author confirms being the sole contributor of this work and approved it for publication.

## Conflict of Interest Statement

The author declares that the research was conducted in the absence of any commercial or financial relationships that could be construed as a potential conflict of interest.
